# Rhythmic fluctuations of saccadic reaction time arising from visual competition

**DOI:** 10.1038/s41598-018-34252-7

**Published:** 2018-10-26

**Authors:** Samson Chota, Canhuang Luo, Sébastien M. Crouzet, Léa Boyer, Ricardo Kienitz, Michael Christoph Schmid, Rufin VanRullen

**Affiliations:** 1 Université de Toulouse, UPS, Centre de Recherche Cerveau et Cognition, 31052 Toulouse, France; 2 CerCo, CNRS UMR 5549, 31052 Toulouse, France; 3 Ernst Strüngmann Institute (ESI) for Neuroscience in Cooperation with Max Planck Society, Deutschordenstrasse 46, 60528 Frankfurt, Germany; 4 Institute of Neuroscience, Newcastle University, Framlington Place, Newcastle upon Tyne, NE2 4HH UK

## Abstract

Recent research indicates that attentional stimulus selection could be a rhythmic process. In monkey, neurons in V4 and IT exhibit rhythmic spiking activity in the theta range in response to a stimulus. When two stimuli are presented together, the rhythmic neuronal responses to each occur in anti-phase, a result indicative of competitive interactions. In addition, it was recently demonstrated that these alternating oscillations in monkey V4 modulate the speed of saccadic responses to a target flashed on one of the two competing stimuli. Here, we replicate a similar behavioral task in humans (7 participants, each performed 4000 trials) and report a pattern of results consistent with the monkey findings: saccadic response times fluctuate in the theta range (6 Hz), with opposite phase for targets flashed on distinct competing stimuli.

## Introduction

Many exploratory actions such as eye movements reveal a specific rhythmicity upon closer inspection. During overt saccadic exploration of the visual field, saccades occur approximately every 200 ms i.e. at ~5 Hz^1–3^. Moreover, even in the absence of eye movements, target detection rates have been shown to vary as a function of the cue-target interval at a similar frequency. It has been proposed that these behavioral fluctuations emerge from rhythmic attentional processes in the theta range^4–9^. Spatial covert attention paradigms have suggested that multiple objects in the visual field are rhythmically and sequentially sampled and that these attentional sampling rhythms are related to brain oscillations in the 4–8 Hz range^10–12^. In addition, causal evidence was provided by Dugué, Marque & VanRullen^13^ who used non-invasive brain stimulation, demonstrating that stimulus processing is vulnerable to disturbances via single TMS pulses at constant intervals in the theta range.

In a recent study, Kienitz *et al*.^14^ linked theta oscillations in macaque V4 to an attentional sampling process^14^. V4 has been previously related to attention, e.g. via lesion studies^15^. Furthermore, sporadic theta oscillations have been measured in V4 as well as in inferotemporal cortex when the animals were viewing a single stimulus^16–20^; when two stimuli were shown together, the competition between them resulted in intricate theta-band oscillatory phase relations between the corresponding IT neural responses^21^. Building on these findings, Kienitz *et al*.^14^ showed that the presence of two visual objects, one in the excitatory center (“object”) and one in the inhibitory surround (“flanker”) of a V4 neuron’s receptive field (RF), resulted in theta-rhythmic multi-unit-activity (MUA). Furthermore, they showed that the saccadic reaction times to targets presented in either of the two stimuli were subject to similar fluctuations at 3–6 Hz. Most importantly, the phase of both RT time-series and MUA oscillations depended on the order of display onset between the object and flanker stimuli. The authors demonstrated that these theta-rhythmic fluctuations emerge from competitive receptive field interactions, and could at least partially underlie the rhythmic attentional sampling of multiple objects observed in numerous human studies^10–12^.

In the current study, we investigate this question in human subjects, using a behavioral spatial attention paradigm directly inspired by this monkey study^14^. A central disk object and two bar flankers were presented in the periphery with asynchronous sequential onset (500 ms SOA), and remained on the screen while participants maintained fixation. After a varying SOA following the second stimulus onset, a target was presented either in the central object or the flankers. The subjects were instructed to perform a saccade to the target. Reaction times was investigated as a function of the SOA between the second stimulus and the target. As found in monkeys, the analysis of RT time-series revealed an oscillation at approximately 6 Hz; furthermore the phase of this oscillation was dependent on both the initial stimulus order (object- or flanker-first) and on the location of the target (object or flanker).

## Results

**Figure 1:**
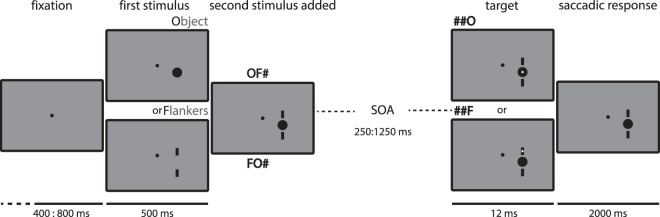
Experimental Protocol. Trials started with a fixation period during which participants maintained fixation for a variable delay between 400 to 800 ms. Following the fixation period the first stimulus (object or flanker) was presented for 500 ms after which a second object (flanker if object first (OF#) and vice versa (FO#)) was added. After a variable SOA of 250 to 1250 ms (in steps of 12 ms) a target was presented for 12 ms, either in the center of the object (##O) or in the flanker (##F). Participants were instructed to respond to the target with a saccade towards the stimulus in which it appeared.

In the current experiment we investigated these fluctuations in a target detection task in humans, directly mirroring one part of the monkey study by Kienitz *et al*.^14^. We presented a first stimulus, to which a second one was added (Fig. 1). A target was then presented in either of the two objects with many possible SOA’s, spanning a 1000 ms interval in 12 ms steps. The participant was instructed to make a saccade to the target as fast as possible. We analyzed the reaction times as a function of the variable SOA. The dense temporal sampling of the target interval allowed us to quantify these behavioral modulations using frequency decomposition methods (Fourier and Hilbert transforms).

### Fluctuations in RT time-series

**Figure 2:**
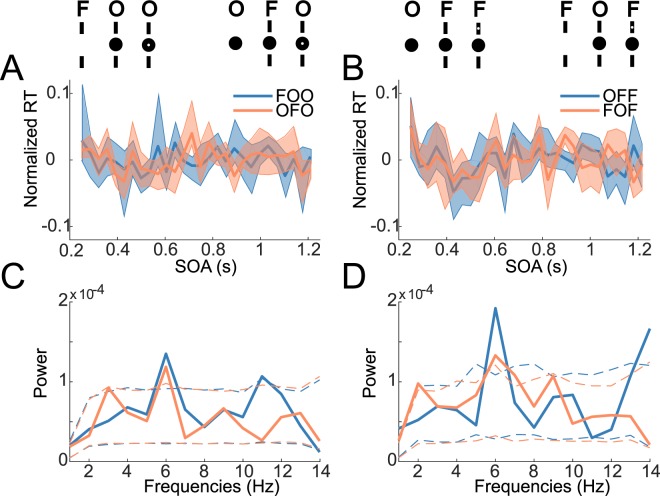
Analysis of RT fluctuations for each condition. The four conditions are illustrated at the top for reference; they vary based on the order of presentation of the stimuli (Object-first or Flanker-first) as well as the site of target presentation (Object, Flanker). (**A**,**B**) RT time-series averaged across subjects (error bars indicate bootstrapped 95% CI) for each of the four conditions, grouped according to the site of target presentation (Object in A, Flanker in B). (**C**,**D**) Average power spectrum across subjects for each of the four conditions (grouped as previously). Dotted lines indicate the bootstrapped 95% confidence interval under the null hypothesis that all frequencies have similar power. We observed a significant peak at 6 Hz for all four sequence types.

To investigate rhythmic fluctuations in behavior, we performed a frequency analysis on the RT time-series for each condition separately. Although the average time courses (Fig. 2A,B) do not show evident oscillations (possibly due to small differences in phase or frequency across subjects), the power spectra revealed a dominant oscillation at around 6 Hz for all conditions (Fig. 2C,D). To test if the observed peak at this specific frequency could be due to chance, we created 2000 surrogates by shuffling the 28 SOA-bin labels within subjects and within conditions and recalculating the power spectra. P-values were computed as the percentile of the mean power values within the bootstrapping distribution. This allowed us to test the null-hypothesis that all frequencies share similar power content. Both main data as well as the surrogates were de-trended. For all four conditions the observed spectral peak at 6 Hz proved to be significantly higher compared to the surrogate distribution (Fig. 2C,D; FOO: p = 0.0045, OFO: p = 0.0012, OFF: p < 0.0005, FOF: p = 0.0255). We observed additional significant peaks at 11 Hz (FOO: p = 0.043), 7 Hz (FOF: p = 0.0185), 14 Hz (OFF: p = 0.0095) as well as 1 Hz (OFF: p = 0.003; FOF: p = 0.021). As these additional effects were not consistent across the four conditions, we did not explore them further. The 6 Hz spectral peak, however, was present in all four conditions. Notably, the likelihood of all four conditions showing a significant peak at the same frequency would be extremely small under the null hypothesis: if the probability of one given frequency exceeding the statistical threshold is 0.05, then the likelihood of this event happening 4 successive times at the same frequency is 14 (frequencies) * 0.05^4^ (conditions) = 0.0000875, i.e. p < 0.0001. We found no significant effect of stimulus sequence on the number of saccades during catch trials.

### Opposed sequences reveal anti-phasic RT fluctuations

The spectral analysis illustrated in Fig. 2 reveals that all 4 experimental conditions display significant 6 Hz oscillations in behavioral RT time courses. Do all 4 oscillations share the same phase, or does the phase differ depending on task factors? In order to analyze potential differences in the phase of the observed oscillations, we subtracted the RT time-series of conditions that had identical target locations. These conditions differed only in the history of object and flanker presentation times. Stimulus competition normally begins when the second object appears on the screen; according to the idea of rhythmic attention sampling, this competition would initially be biased towards the second object (the last one to appear), then attention would move on to sample the first, and rhythmically alternate between them on subsequent cycles^8,21–23^. In other words, the phase of attention sampling (and thus the phase of behavioral RT oscillations, for a fixed target location) should be opposite for Object-first and Flanker-first sequences. Such an anti-phasic relationship should be visible as an elevated peak in the power spectrum of the difference in the RT time-series. Conversely, if oscillations for the two conditions shared the same phase, the subtraction should reduce the amplitude of the 6 Hz spectral peak.

**Figure 3:**
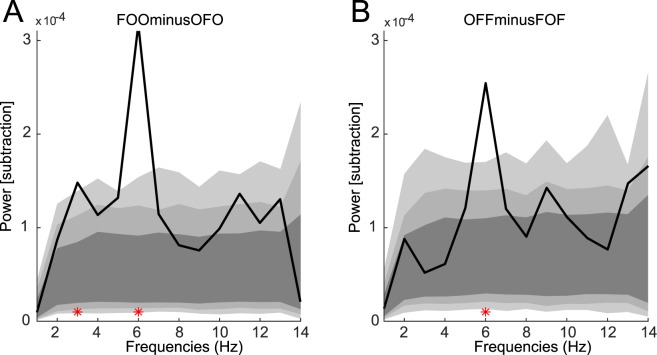
Frequency Analysis of the difference in RT time series between conditions with identical target location (FOO vs. OFO in **A**, OFF vs. FOF in **B**). Light grey areas indicates the bootstrapped 99.9% CI (99% in darker grey, 95% CI in darkest grey). Red dots indicate frequencies with significantly higher power compared to a null hypothesis distribution calculated by randomizing the SOAs within opposing sequences.

As expected according to the rhythmic attention sampling idea, we observed an enhanced spectral peak at 6 Hz for both time-course subtractions (Fig. 3), indicating that the experimental conditions shared a frequency-specific oscillatory component, however with opposite phase for the two stimulation sequences. For both comparisons, the amplitude of the 6 Hz peak in the subtraction was higher than that measured in either of the original signals (compare values in Fig. 3A,B with those in Fig. 2C,D), which is compatible with an anti-phase, but not an in-phase relation between the stimulation sequences. The significance of the 6 Hz peak (FOO minus OFO: p < 0.001, OFF minus FOF: p < 0.001, Bonferroni corrected) was confirmed by comparing it to a null hypothesis distribution, calculated by randomizing a subset of the target SOAs within subjects and between opposing sequences (FOO and OFO, OFF and FOF) 2000 times. The number of reaction times that were taken as the subset was determined by the total number of reaction times recorded for that specific SOA during the experiment, resulting in an identical number of trials per SOA in the original and randomized datasets. This method revealed an additional significant peak at 3 Hz (p < 0.001) for the FOO minus OFO condition, however markedly smaller than the peaks at 6 Hz.

## Discussion

Our experimental paradigm was based on previous primate studies^14,21^ that investigated the oscillatory responses to competing stimuli. The paradigms used in these previous papers were as follows: A sequence of three stimuli was presented, Object followed by Flanker, or vice versa, followed by a target that could appear either in the Object or in the Flanker (Fig. 1). Firing responses of V4 and IT neurons selective for either of the stimuli (Object, Flanker) were recorded. The authors observed that firing rates oscillated at 4–5 Hz following stimulus presentation. Furthermore, if the neuron’s “preferred” stimulus was presented second, the same oscillation was observed but in anti-phase (compared to the condition with “preferred” stimulus first). These oscillations were present but significantly weaker when only one stimulus was presented (even when it was the neuron’s preferred stimulus). Finally, the Kienitz *et al*.^14^ study further revealed direct behavioral correlates of these oscillations, whereby rhythmic fluctuations of saccadic reaction times accompanied the firing rate oscillations. Altogether, these findings imply that the competition between visual neurons coding for distinct neighboring objects in the visual scene is modulated at around 4–5 Hz. The authors suggested that this modulation of competition might reflect a rhythmic attentional sampling mechanism, initiated by the sequential presentation of the two competing objects.

Here we investigated whether a similar mechanism could lead to behavioral theta-rhythmic fluctuations in humans. Using a similar experimental paradigm, we provided strong evidence that all four stimulus sequences (i.e., regardless of stimulus temporal order and target location) lead to rhythmic modulations in saccadic reaction times. Spectral analysis of the RT-time series revealed that 6 Hz is the dominant frequency of these fluctuations. We also confirmed that opposing sequences that differed only in the temporal order of the first two stimuli (Object-first or Flanker-first, keeping target location constant), induced fluctuations with opposing phase. This was shown by subtracting the two RT time-series followed by an examination of the spectral content of the difference wave. Our results are consistent with those of Kienitz *et al*.^14^, supporting the idea that an attentional sampling mechanism may underlie the theta-rhythmic fluctuations in behavior in monkeys and importantly extending them to humans.

An increasing number of studies have reported rhythmic fluctuations in perceptual performance. In the specific case of spatial attention, when multiple objects are presented on the screen, attention seems to alternatingly switch between the attended objects^4–6,8,11,13,24,25^. This alternation is assumed to be instantiated by a sequential attentional sampling process that leads to behavioral oscillations that are out of phase for the different attentional targets. A crucial element in the investigation of attentional processes is the relationship between behavior and neuronal processes. While it is difficult to investigate theta oscillations measured from single cells in humans, there are a number of electrophysiological EEG/MEG studies successfully relating theta rhythmicity in the human brain to attention. Busch & VanRullen^10^ investigated pre-stimulus oscillatory activity, by contrasting trials in which target detection was successful to unsuccessful trials. They found that the pre-stimulus phase in the theta band was predictive of stimulus detection, however only for attended stimuli, indicating that attentional sampling operates at ~7 Hz. Landau *et al*.^26^ reported that pre-target gamma-band activity was modulated at 4 Hz when two stimuli were presented on the screen. Furthermore this modulation was predictive of task performance and provided important evidence for the hypothesis that the presentation of a relevant stimulus resets an ongoing attentional mechanism. These findings are especially relevant in the context of our study, since it is based on the assumption that the behavioral performance fluctuations are a result of a phase reset of an ongoing attentional oscillation by the stimulus onsets in the display sequence.

The above mentioned studies indicate that the pre-stimulus phase of theta oscillations can predict performance^10,11,27^. This raises the question of how ongoing and evoked (phase-reset) theta oscillations interact to influence task performance. Insight can be provided by the findings of Dugue *et al*.^11^: In their EEG experiment on visual search they observed pre-stimulus theta phase opposition between successful and unsuccessful search trials, together with stronger post-stimulus theta phase-locking as well as higher post-stimulus theta amplitude for successful compared to unsuccessful trials. This suggests that the pre-stimulus theta phase was indicative of both the post-stimulus EEG signal and of task performance; to account for this relation between pre- and post-stimulus oscillations, they assumed that the presentation of a stimulus only leads to a partial phase reset of ongoing theta oscillations.

While our paradigm, closely resembling Kienitz *et al*.^14^, was mostly concerned with covert attention, it remains unclear how our findings would translate to overt attention. Would we observe similar periodic fluctuations when observers are free to explore the visual scene? Interestingly eye movements occur approximately every 200–300 ms even in absence of a task, i.e. during free exploration^1^. This rhythmicity is preserved if a target detection task is performed, in which participants can explore the visual scene freely^2^. A crucial piece of evidence in the study by Hogendoorn^2^ is the fact that the phase of the behavioral oscillation did not change as a result of the saccade, indicating that the saccades may have been executed as part of an underlying attentional oscillation. One interpretation of these results is thus that a unique theta-rhythmic sampling mechanism could underlie both overt and covert forms of spatial attention.

Kienitz *et al*.^14^ suggested that center surround interactions between neighboring stimuli in V4 might facilitate attentional stimulus selection. The fact that the excitatory center of V4-neurons found by Kienitz *et al*.^14^ showed maximal responses for stimuli measuring 2° of visual angle certainly restricts the conclusions that can be drawn in terms of larger objects or of wider distances. It is thus an open question how the brain could instantiate attentional selection among objects that are further spread across the visual field. Would this interaction still arise in V4, or in hierarchically higher areas with larger receptive field sizes spanning larger distances? It has been shown that similar behavioral competition can result in anti-phase theta-band rhythmic attentional sampling between two stimuli presented in opposite hemi fields^4–6^, and that corresponding neural correlates can be observed in visual cortex (based on MEG source reconstruction)^26^. The precise neural source of this large-scale rhythmic attentional sampling, however, remains to be determined by direct electrophysiological experiments.

Our paradigm used 3 stimuli (one object and 2 flankers) of which 2 (the object and one flanker) were behavioral significant. It would therefore be highly interesting to investigate how attention behaves if the number of potential target positions is extended beyond 2. Similar experiments have been conducted by Holcombe and Chen^7^ as well as Macdonald *et al*.^24^. They suggest an attentional sampling mechanism with limited capacity, such that multiple objects are sampled less and less frequently with increasing numbers of objects. We intend to investigate this matter in future experiments.

In conclusion we provided new evidence for a sequential attentional sampling mechanism in the theta range (6 Hz) in humans. Our findings support those of Kienitz *et al*.^14^, and demonstrate similar behavioral patterns in monkeys and humans. Our conclusions provide further insight into how the brain resolves potential competition by using attentional mechanisms to rhythmically select relevant stimuli.

## Materials and Methods

### Participants

Seven volunteers (aged 19–25, 3 females, all right-handed) with normal or corrected to normal vision participated in the experiment. Informed consent forms were signed before the experiment. The experiment was carried out in accordance with the protocol approved by the Centre National de la Recherche Scientifique ethical committee and followed the Code of Ethics of the World Medical Association (Declaration of Helsinki).

### Protocol

Stimuli were presented at a distance of 50 cm with a cathode ray monitor (1280 × 1024 resolution, 85 Hz refresh rate) using the Psychophysics Toolbox^28^ running in MATLAB (MathWorks). Eye movements were recorded and monitored online using an EyeLink 1000 Desktop Mount (SR Research). A 9-point calibration was performed before each block of trials. Throughout the Methods section we will explicitly note the differences with the monkey study by Kienitz *et al*.^14^. If no mention is provided, task parameters at hand were kept identical.

Stimuli consisted of a fixation dot (central black dot, diameter = 0.3°; [0.07° in Kienitz *et al*.^14^]), the object (a black disk in the lower right part of the screen, 2° diameter, positioned 4° right and 2° down from fixation center [in Kienitz *et al*.^14^ the disk was positioned, within the RF of a V4 neuron]) and the flankers (two bars above and below the disk, height = 1°, width = 0.25°, 1° gap between disk and each bar) (Fig. 1). Stimuli were presented on a gray background.

After a pseudo-random (400–800 ms [1000 ms in Kienitz *et al*.^14^]) interval during which participants maintained central fixation, the first stimulus of the sequence, either the Object or the Flankers, appeared. We introduced a variable delay in this pre-stimulus interval to counteract potential attentional effects that could be introduced by the predictability of the stimulus onset. The second stimulus (the Flankers if the Object was presented first and vice versa) was added to the display 500 ms later [Object was always presented first in Kienitz *et al*.^14^]. After another variable SOA (250 to 1250 ms, in steps of 12 ms [0 to 750 ms, in steps of 37.5 ms in Kienitz *et al*.^14^]) a small target (0.2° diameter white dot) then appeared for a single frame (12 ms [8.3 ms in Kienitz *et al*.^14^]), either in the center of the upper flanker or the center of the disk object. In one out of 16 trials ([1 out of 3 in Kienitz *et al*.^14^]), no target was presented (catch trials). This reduction in the number in catch trials still allowed us to reliably control for non-target related saccades.

For consistency and clarity we will now refer to the four possible (non-catch) trial types as the conditions: OFO, OFF, FOO, FOF, where the 1st letter denotes the first stimulus onset (O for object, F for flanker), the 2^nd^ letter corresponds to the second stimulus presented (identical notation), and the third to the stimulus in which the target appeared (identical notation) (See upper part of Fig. 2).

The timing of the target relative to the onset of the second stimulus (so-called stimulus onset asynchrony or SOA), was drawn from a uniform distribution with 85 steps from 250 to 1250 ms (corresponding to the refresh rate of the screen). The SOA tested on each trial was optimized online to equalize the number of trials obtained for each of the 85 SOA conditions: if a trial was not valid (see below), the same SOA value was more likely to be tested again.

The luminance of the target was adapted separately for each condition using a QUEST procedure^29^ so that each participant’s detection performance remained around 90%. We chose the 90% threshold in order to collect sufficient trials that could be entered in our analysis and at the same time require participants to pay attention. Because of the high number of trials, involving multiple sessions over several days, the luminance value defined by the QUEST procedure was adapted dynamically, based only on the last 40 trials.

Participants had to respond to the target by performing a saccade towards it. A saccade was considered valid if it landed in one of two 2° square boxes centered respectively on the object and upper flanker. Only valid saccades were included for further analysis. Reaction times (RT) were defined as the time between target onset and the time at which the eye position left a fixation region of 1° radius around the fixation point. Saccade durations were defined as the interval between the eye leaving the fixation region and the eye landing on one of the two boxes centered on object or flanker. To encourage participants to respond as fast as possible, online RT measurement were used and a message saying “TOO SLOW” was displayed if the RT of a given trial was above 2.5*SD from average (the average was calculated based only on valid trials (see below)). The experiment was stopped automatically when a participant obtained 4000 valid trials (split in blocks of 64). A trial was considered valid if (1) a target was presented, (2) the participant made a valid saccade to the correct location and (3) the RT was not an outlier (limits corresponding to 2.5*SD, updated online after each trial by considering all previous trials, even non-valid ones).

### Data Analysis

During preprocessing, we removed all trials in which the saccade duration was above 70 ms, or the luminance value selected by the QUEST was outside the 2.5*SD limits (across all luminance values tested). For the RT analysis, we considered only trials in which a target was present (15 out of 16 trials) and the saccade was made to the correct location. This resulted in the inclusion of 72.67% of all trials on average (±5.62% standard error of mean across subjects).

To increase the number of trials per bin, SOA values were binned in groups of 3 (resulting in a change of effective sampling frequency from 85 Hz to 28 Hz). We validated in a separate re-analysis (not detailed here) that the exact position of the bin limits did not affect any of our findings. All the results were analyzed based on the binned SOA values. Single-trial RT values were then aggregated for each condition and SOA bin, outliers were removed (values outside 2.5 SD around average), and averages computed to obtain RT time-series (average RT as a function of SOA) for each of the 4 types of sequences (OFO, OFF, FOO, FOF) and each subject.

We observed a strong negative trend in the RT time-series: early SOAs resulted in much slower RTs for most subjects. We attribute the slow RT decrease to the hazard rate although an additional forward masking effect could contribute to the early RT’s (~200 ms). To minimize the influence of these factors, RT time-series were de-trended using a second order polynomial (as is commonly done in most previous studies investigating oscillations of behavioral measures)^6,9,14^.

### Frequency Analysis

RT time-series were analyzed in the frequency domain using both FFT and Hilbert methods. The 28 SOA values over a one second window allowed for a Nyquist frequency of 14 Hz. The complex FFT coefficients were squared to obtain oscillatory power at each frequency (Figs 2C,D and 3C,D).

## Electronic supplementary material

Supplementary results
