# Supplementary material for: Rhythmic fluctuations of saccadic reaction time arising from visual competition
Source: Sci Rep. 2018 Oct 26;8:15889. doi: 10.1038/s41598-018-34252-7 (PMC6203856; doi:10.1038/s41598-018-34252-7)
Supplement: Supplementary file 1 — Supplementary results [file 41598_2018_34252_MOESM1_ESM.docx]

**Title: Rhythmic fluctuations of saccadic reaction time arising from visual competition**

Samson Chota^1,2^*, Canhuang Luo^1,2^*, Sébastien M. Crouzet^1,2^, Léa Boyer^1,2^, Ricardo Kienitz^3,4^ ,
Michael C. Schmid^3,4^, and Rufin VanRullen^1,2^

^1^Université de Toulouse, UPS, Centre de Recherche Cerveau et Cognition, 31052 Toulouse, France

^2^CerCo, CNRS UMR 5549, 31052 Toulouse, France

^3^Ernst Strüngmann Institute (ESI) for Neuroscience in Cooperation with Max Planck Society, Deutschordenstrasse 46, 60528 Frankfurt, Germany

^4^ Institute of Neuroscience, Newcastle University, Framlington Place, Newcastle upon Tyne, NE2 4HH, UK

*equal author contribution

Figure 1. Individual RT fluctuations. First Row: non-detrended RT time-series (red dots) and square fit (black line) that was subtracted. Second Row: de-trended RT time-series (red dots). Goodness of fit (GOF), was calculated by correlated the original data with the fitted (sine fit) data (black line). The frequency of the fitted sine wave was fixed at 6 Hz.

Figure 2, Individual RT fluctuations, after subtraction (FOO minus OFO, OFF minus FOF, red dots). Goodness of fit (GOF), was calculated by correlated the original data with the fitted (sine fit) data (black lines). The frequency of the fitted sine wave was fixed at 6 Hz.
